# Beyond A1c: Redefining Evidence and Policy to Advance Therapies in Type 1 Diabetes Care

**DOI:** 10.17925/EE.2026.22.1.4

**Published:** 2026-02-13

**Authors:** Huajing Ni, Jerry R Greenfield, Jennifer R Snaith

**Affiliations:** 1. Victor Chang Cardiac Research Institute, Darlinghurst, Sydney, Australia; 2. St. Vincent’s Healthcare Campus, School of Clinical Medicine, Faculty of Medicine and Health, University of New South Wales, Sydney, Australia; 3. Department of Diabetes and Endocrinology, St. Vincent’s Hospital, Darlinghurst, Sydney, Australia

**Keywords:** Adjunctive therapies, cardiometabolic risk factors, clinical endpoints, diabetes distress, glycaemic control, government regulations, patient-reported outcome measures (PROMs), type 1 diabetes

## Abstract

Innovation in technology has revolutionized modern-day type 1 diabetes (T1D) care, significantly improving glycaemic control and the patient experience. However, the outcome measures used in clinical trials, which inform policies, remain stagnant. Progress has been made in the search for adjunctive therapies beyond insulin to reduce residual cardiometabolic risk. The importance of patient-centred strategies is also increasingly recognized. The future of T1D research calls for the adaptation by regulators to accept additional endpoints beyond haemoglobin A1C (HbA1c).

The past century has witnessed substantial evolution in the assessment and management of type 1 diabetes (T1D), paralleling advances in our understanding of the condition and in innovation in technology. Since the ground-breaking discovery of insulin in the 1920s, T1D has been transformed from a fatal disease to a manageable chronic condition. The introduction of long- and rapid-acting insulin analogues, insulin pumps and glucose monitoring technology has revolutionized glycemic control, while recent innovations in continuous glucose monitoring (CGM) sensors and semi-automated algorithms have transformed T1D management by enabling dynamic glucose tracking, carbohydrate counting and flexible insulin dosing as standard practice.^1^ Although treatment options have vastly improved, insulin-only therapy remains the mainstay of care in the modern-day management of T1D, and residual cardiometabolic risk remains unacceptably high in the T1D population. This unmet need underscores the necessity for adjunctive strategies that offer benefits beyond glucose control.^2^

The Diabetes Control and Complications Trial (DCCT; ClinicalTrials. gov identifier: NCT00360815) established a glucocentric dogma in T1D care by demonstrating that intensive glycaemic control and tight haemoglobin A1C (HbA1c) targets translated into a significant reduction in long-term risks of cardiovascular complications.^3^ However, the intensification of insulin therapy was unfortunately accompanied by trade-offs including higher rates of severe hypoglycaemia and weight gain, leading to increased cardiometabolic risk.^4^ In the 21st century, standardized CGM reports have become the cornerstones of real-world glucose monitoring, supplementing glucometers. While HbA1c targets remain a foundation of recommended practice, the incorporation of CGM-derived endpoints, including time-in-range, time-above-range and time-below-range, facilitates a more holistic evaluation of metabolic control. They provide easily interpretable and individualized glucose metrics, which can be used to inform decisions regarding therapy adjustments and to achieve intensive glucose targets informed by the DCCT. In addition to providing a more nuanced portrait of day-to-day glucose variability by capturing glucose excursions, these metrics are now routinely used to support shared decision-making between patients with T1D and clinicians. However, CGM metrics are yet to be accepted by the Food and Drug Administration (FDA) as a clinical trial endpoint for a glycaemia-based indication for new therapies in T1D.^5^ Instead, the reliance on HbA1c persists, leaving a fundamental challenge in modern T1D care – defining meaningful clinical endpoints that capture the multidimensional nature of the condition.

The search for adjunctive non-insulin therapies has extended beyond glycaemia to target weight management, insulin resistance and cardiometabolic risk reduction.^6^ Sodium–glucose cotransporter 2 inhibitors, glucagon-like peptide-1 receptor agonists, with or without glucose-dependent insulinotropic polypeptide co-agonism, represent the most studied therapeutic classes and are showing great promise to complement insulin by mitigating factors that contribute to adverse cardiovascular risk profiles. Early and on-going research in this space seeks to find the balance between efficacy and safety, tolerability and cost of such therapies, as well as patient acceptability, which is key to long-term adherence, and will be critical to the realization of any meaningful reduction in morbidity and mortality. The pursuit of such therapies represents a major paradigm shift in how the global diabetes community is conceptualizing T1D management, moving towards a more holistic approach.

In addition to metabolic outcomes, emotional and psychosocial aspects of T1D now play a critical role in overall wellbeing. Diabetes distress, the emotional burdens that arise as a direct consequence of living with T1D, is a growing source of concern for patients with T1D, caregivers and clinicians alike. It results from the stress of on-going glucose monitoring, dietary restrictions and fear of long-term complications.^7^ This distress can further impact self-care behaviours, treatment adherence and engagement with healthcare services, with downstream effects on clinical outcomes.^8^ Strategies to help address diabetes distress and to support patients with T1D in their disease journey include comprehensive care models that integrate medical management with psychosocial support, patient education and individualized strategies to help enhance resilience and achieve a sense of empowerment. Diabetes distress should be routinely assessed as part of a holistic approach to T1D care. In this regard, a recent European Medicines Agency workshop explored ways to better integrate patient experience data into regulatory processes.^9^ The outcome of this workshop formally expands registry data beyond traditional clinical endpoints, recognizing the value of patient-driven, real-world evidence. Despite this encouraging step forward, challenges remain in the alignment between registry holders, regulators and industry of incorporating patient experience data in the approval processes.^9^

The future of T1D care calls for the integration of technology, physiology and psychology and invites a broader research agenda that focuses on measures of outcomes beyond glucose and is more centred around patient-reported priorities, particularly around diabetes distress. The importance of people living with T1D in defining and determining the focus of future research pathways cannot be understated. Regulators also need to evolve with this major transformation in diabetes care and accept trial outcomes beyond HbA1c. Technological innovations have undoubtedly improved the precision and convenience of glucose management. However, translating these advances into tangible reductions in adverse outcomes will require robust and long-term studies that evaluate not only glycemic control but also other important clinical endpoints, such as body weight and composition, lipid profiles, cardiovascular events and health-related quality of life. Such evidence will be crucial in informing guidelines and shared decision-making practices that align with patient preferences and clinical priorities.
